# Effects of high salinity in drinking water on behaviors, growth, and renal electrolyte excretion in crossbred Boer goats under tropical conditions

**DOI:** 10.14202/vetworld.2022.834-840

**Published:** 2022-04-06

**Authors:** Nguyen Thiet, Nguyen Van Hon, Nguyen Trong Ngu, Sumpun Thammacharoen

**Affiliations:** 1 Department of Agricultural Technology, College of Rural Development, Can Tho University, 3/2 Street, Can Tho City 94000, Vietnam; 2 Department of Veterinary Medicine, College of Agriculture, Can Tho University, 3/2 Street, Can Tho City 94000, Vietnam; 3 Department of Physiology, Faculty of Veterinary Science, Chulalongkorn University, Henri Dunang Street, Bangkok 10330, Thailand

**Keywords:** electrolytes, drinking behavior, goat, kidney, saline water

## Abstract

**Background and Aim:**

The high salinity of drinking water has been a significant problem of the Mekong Rivers Delta. Animals drinking high salinity water altered feed and water intake (WI), urinary electrolytes excretion, and productivity. This study aimed to evaluate the effects of high salinity in drinking water on drinking and eating behaviors and kidney function in crossbred goats.

**Materials and Methods:**

The experiment was completely randomized with two treatments: freshwater (0%, seawater [SW0]) and water high in salinity (1.5%, SW1.5) from diluted SW, with five replicates (five animals per treatment). This experiment lasted 3 weeks: the 1^st^ week for the pre-treatment period and the 2^nd^-3^rd^ weeks for the post-treatment. Dry matter intake (DMI) and WI were recorded every day, while urine volume (UV) was determined from day 8 to day 21. Blood and urinary samples were collected on days 6, 14, and 21 of the study for electrolytes and creatinine analysis.

**Results:**

The results demonstrated that both DMI and WI were affected by SW1.5 (p<0.05). Goats drinking from SW1.5 had lower DMI during D19–21, and the ratio of DMI/WI was significantly different during D16–21 (p<0.05). Interestingly, the UV from SW1.5 was higher than that from SW0 during D13–21 (p<0.05). Although the body weights (BW) of both groups were similar (p>0.05), the weight gain observed in the SW1.5 group tended to decrease (p=0.056) at the 2^nd^ week. The concentration of electrolytes in blood did not differ between the groups (p>0.05). In contrast, the concentration and excretion of Na+ and Cl- in urine increased in SW1.5 goats at D14 (p<0.05), while creatinine levels in the blood remained normal (p>0.05).

**Conclusion:**

The study concluded that crossbred male goats can tolerate 1.5% saline water from diluted SW for 2 weeks. The high salinity in water influences drinking and eating behavior in growing goats. However, the adaptive mechanism by increasing urine output and reducing the reabsorption of Na+ and Cl- in the kidney is the key function and works faster than behavioral responses. The kidney apparently drives drinking behavior during high salinity water consumption.

## Introduction

Vietnam is classified as a water-deficit country in which the mean surface water and groundwater is 4400 m^3^/person/year (compared to 7400 m^3^/person/year in the world on average). According to calculations, if the sea level rises 1 m, 39% of the Mekong River Delta (MRD) area becomes at risk of salinity intrusion. In 2016, in the coastal provinces in the MRD, the salinity levels in some locations ranged from 0.6 to 1.5%. Interestingly, Boer crossbred goats are the predominant breed and are well developed in this area. The previous study found that goats are more tolerant to high saline in drinking water than sheep [1]. Goats could accept 1.5% NaCl in drinking water [2]. Saanen crossbred goats and growing local goats in Vietnam rejected saline water at 1.5-2.0% [3]. Boer goats rejected saline water at 1.25-1.5% NaCl and were more sensitive to the ingestion of salt from drinking water after prolonged exposure to saline water [4]. However, the previous study reported that goats can tolerate saline water up to 2% for at least 2 weeks [5].

In this context, the animals’ ability to tolerate various degrees of salt loads in drinking water may be related to kidney function [6]. Due to the kidney’s important role in regulating body fluids and compositions, some studies have suggested that sheep drinking water containing 1.3% NaCl appear to develop an adaptation for salt tolerance without any negative effects [7]. This ability came from a renal adjustment that increased filtration and eliminated salt [6]. Similarly, Abou Hussien *et al*. [8] found that sheep and goats drinking saline water controlled their salt load by excreting more urine and increasing the filtration rate, while camels drank less saline water to decrease salt stress. Although goats may be tolerant to saline water up to 1.5% due to renal adjustment, difference in breeds, or climate conditions as reported by previous studies, there is little information about the chronological responses of Boer crossbred goats to diluted seawater (SW) under tropical conditions.

Therefore, this study aimed to investigate the effects of high salinity in drinking water on chronological behaviors and renal electrolyte excretion responses in growing crossbred goats under tropical conditions. For this purpose, we hypothesized that Boer crossbred goats may adapt to high salinity in drinking water with either chronological behaviors or changes in renal electrolyte excretion.

## Materials and Methods

### Ethical approval

The study was approved by the Scientific Committee, Can Tho University (#3559), Vietnam.

### Study period and location

The study was carried out from September 2020 to March 2021 at the Experimental Farm, College of Rural Development, Cantho University, Vietnam. The samples were analyzed at Department of Animal Science, College of Agriculture, Cantho University, Vietnam.

### Experimental design and animal care

The experiment was conducted on 10 male crossbred Boer goats (Boer×Bach Thao goats, 7-8 months old) with an average body weight (BW) of 22.70±0.30 kg. In this study, we used the male Boer crossbred goats because the farmers prefer the male goats for meat purposes due to better daily weight gain than female Boer crossbred goats. Each animal was in a healthy state and was not suffering from dangerous infectious diseases. All animals were kept in individual metabolic cages in 1.2×0.7 m shaped pens with plastic floors, feeders, and drinking troughs for the adaptation period (10 days). During this period, all goats ate the same experimental diets and drank freshwater. Afterward, the experiment was completely randomized with two groups: a control group of goats drinking freshwater (SW0) and a treatment group of goats drinking saline water with a concentration of 1.5% (SW1.5). The present study has only five replicates, and this is a limitation. However, we think that this sample size has enough power and proved consistent with our previous studies [9,10].

The study used concentrated SW (9%) mixed with freshwater to achieve water with a salt concentration of 1.5% (SW1.5), according to the formula C_1_V_1_=C_2_V_2_ (where C_1_ is the concentration of the starting solution; V_1_ is the volume of the starting solution; C_2_ is the concentration of the final solution; and V_2_ is the volume of the final solution), and the salinity was then checked by a refractometer (Master S28M, Atago, Japan). We bought the concentrated SW from an aquaculture farm and delivered it to the experimental site. The water samples from the SW0 and SW1.5 groups were analyzed for chemical composition, as shown in Table-1. This experiment lasted for 3 weeks, with 7 days for the pre-treatment period (from the 1^st^ to 7^th^ day) and 14 days (from the 8^th^ to 21^st^ day) for the treatment period. All goats were offered the same rations containing 70% corn silage and 30% concentrate (consisting of 8% rice bran, 11.3% corn meal, 7.8% soybean meal, 0.9% limestone, and 2% molasses) formulated as total mixed rations (TMR) according to the recommendation of NRC [11]. The ingredients and chemical compositions of the rations are presented in Table-2. The goats received TMR *ad libitum* twice daily at 07.00 and 14.00 h and had free access to water.

**Table 1:** The compositions of freshwater and high salinity water.

Items	Freshwater (SW0)	High saline water (SW1.5)
EC (mS/cm)	0.28	33.00
TDS (g/L)	0.127	15.00
Cl^-^ (g/L)	0.028	8.77
K^+^ (mg/L)	4.35	156.00
Na^+^ (mg/L)	16.60	4412.00
Ca^2+^ (mg/L)	15.50	92.30
Mg^2+^ (mg/L)	9.91	606.00

EC=Electrical Conductivity; TDS=Total Dissolved Solids; Cl^-^=Chloride; Na^+^=Sodium; K^+^=Potassium; Ca^2+^=Calcium and Mg^2+^=Magnesium, SW=Seawater

**Table 2:** Ingredients and chemical composition of the TMR.

Ingredients	% DM
Corn silage	70.00
Rice bran	8.00
Corn meal	11.30
Soybean meal	7.80
Limestone	0.90
Molasses	2.00
Chemical composition	
Dry matter	29.50
Crude protein	16.20
Ether extract	2.01
Acid detergent fiber	28.50
Neutral detergent fiber	39.50
Ash	9.70
Na	0.06
K	1.71
Cl	1.06

Na^+^=Sodium; K^+^=Potassium; Cl^-^=Chloride. TMR=Total mixed rations

### Meteorological data

The temperature and humidity were recorded at 07.00 h, 09.00, 11.00, 13.00, 15.00, 17.00, and 19.00 h. The temperature and humidity index was calculated as recommended by Thammacharoen *et al*. [12]. The environmental conditions from the current study are presented in Table-3.

**Table 3:** Environmental conditions from the current experiment.

Time	Ambient temperature (°C)	Humidity (%)	THI
07.00	27.88±0.11	81.50±1.13	69.42±0.09
09.00	28.00±0.37	79.25±0.22	69.52±0.03
11.00	29.75±0.56	74.00±1.75	70.95±0.96
13.00	30.31±0.59	71.63±3.01	71.25±0.48
15.00	29.50±0.26	75.25±3.31	70.74±0.21
17.00	28.75±0.43	75.75±2.05	70.71±0.35
19.00	28.25±0.43	78.63±0.96	69.30±0.35

THI=Temperature and humidity index

### Data collection and measurement

Feed offered and feed refusals (9%) were recorded daily in the morning starting from day 1 to the end of the experiment. Daily dry matter intake (DMI) was calculated by the following formula:

Daily feed intake=feed offered-feed refusals (dry matter basis)

Feed and refusal samples were collected daily from day 1 to day 21 and were divided into two parts: one half was immediately dried in the oven at 105°C until its weight remained constant to determine the dry matter (DM), and the remaining samples were kept frozen at −20°C until chemical analysis. At the end of the experiment, all feed samples were thawed and mixed thoroughly, and subsamples were dried at 65°C overnight (approximately 12 h) for nitrogen and ash analysis according to Association of Official Analytical Chemists [13], neutral detergent fiber (NDF), and acid detergent fiber using the procedure developed by Van Soest *et al*. [14]. Sodium (Na^+^), potassium (K^+^), calcium (Ca^2+^), and magnesium (Mg^2+^) were measured by atomic absorption spectrophotometry (Thermo iCE 3000 series, Thermo Fisher Scientific, China), chloride (Cl^-^) was determined by colorimetric titration, sulfate (SO_4_^2-^) was measured by spectrophotometry (Ultraviolet-visible 1800 Shimadzu, Japan), electrical conductivity (EC) was determined using an EC meter (Schott instruments D-55122, Mainz, Germany), and total dissolved solids (TDS) was measured with a refractometer (Master S28M, Atago, Japan). Water intake (WI) was measured daily from the beginning to the end of the experiment by subtracting the weight of water offered from the weight of water refused. All of the goats were weighed before the morning feeding on days 0, 7, 14, and 21 of the experiment, and weight gain was calculated using the following formula:


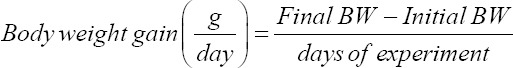


On days 6, 14, and 21, blood samples (4 mL) from the jugular vein were collected at 07.00 before the morning feeding and were then placed in a lithium heparin tube, kept on crushed ice, and centrifuged at 900 x g for 10 min. The plasma samples were stored at −20°C until analysis. In addition, total urine was recorded from day 6 to day 21. The urine samples (50 ml each) from pre-treatment (day 6), day 14, and day 21 were collected, filtered through two layers of cheesecloth, and later analyzed. Plasma creatinine was determined with an automatic clinical chemistry analyzer (XL200, Erba Mannheim, Germany). Plasma and urine electrolytes were measured with an automatic analyzer (ST200 PRO, Sensa Core, India). This study also calculated urinary electrolyte excretion (Uex) with the following formula:

Urinary electrolytes excretion (Uex)=Ue × UV

While U_e_: urinary electrolytes, UV: Urine volume.

### Statistical analysis

The data for DMI per BW, WI per BW, and urine per BW were analyzed with repeated two-way analysis of variance. The significance of the main effects was determined by the Bonferroni posttest. Moreover, the data for BW, weight gain, and plasma and urinary electrolytes were compared using an unpaired t-test. Significance was declared at p<0.05, and a tendency was declared at 0.05<p<0.10.

## Results

**Figure-1:**
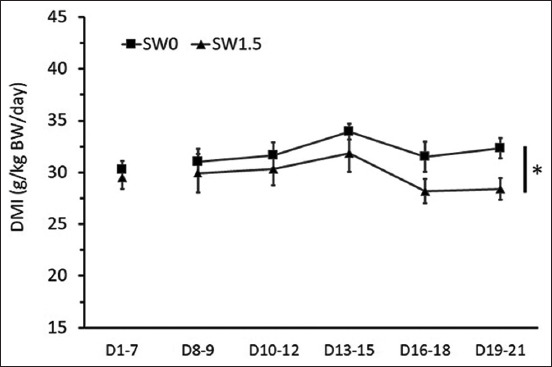
Effects of high salinity in drinking water on dry matter intake (g/kg body weight). D1-7: Pre-treatment from day 1 to day 7; D8-21: Treatment period from day 8 to day 21. *The effect of salinity in drinking water (p<0.05).

**Figure-2:**
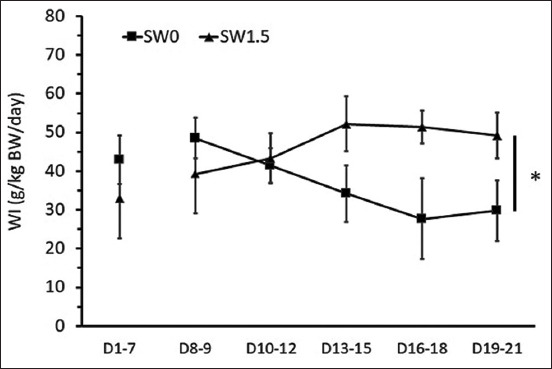
Effects of high salinity in drinking water on daily water intake (g/kg body weight). D1-7: Pre-treatment from day 1 to day 7; D8-21: Treatment period from day 8 to day 21. *The effect of salinity in drinking water (p<0.05).

**Figure-3:**
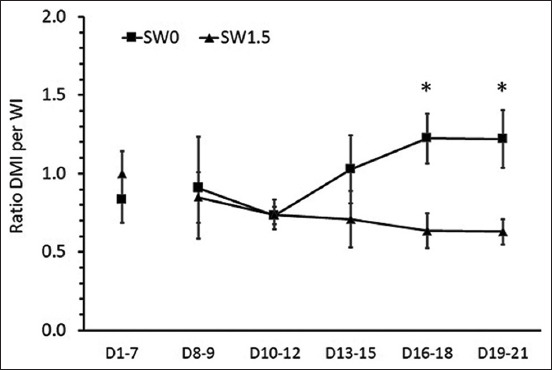
Effects of high salinity in drinking water on ratio of DMI per WI. D1-7: Pre-treatment from day 1 to day 7; D8-21: Treatment period from day 8 to day 21. *The effect of salinity in drinking water (p<0.05).

**Figure-4:**
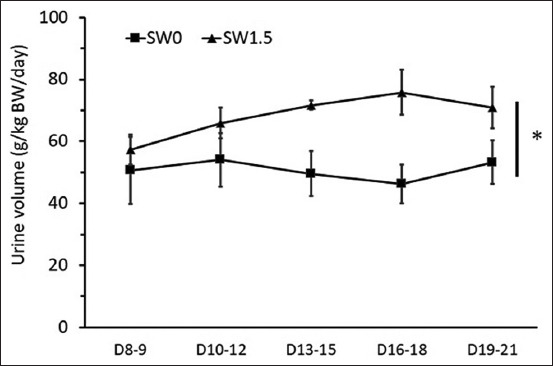
Effects of high salinity in drinking water on daily urine volume (g/kg body weight/day). D8-21: Treatment period from day 8 to day 21. *The effect of salinity in drinking water (p<0.05).

During the pre-treatment period, no differences were detected in the levels of average daily DMI and WI per BW (Figures-1-3). During the 2 weeks of the experimental period, high salinity in drinking water influenced eating and drinking behaviors (Figures-1-3) and urine volume (UV) (Figure-4). The average daily DMI per BW from SW1.5 was lower than that from SW0, especially on D19–21 (Figure-1, p=0.01). Likewise, an effect of high salinity in drinking water was observed on the average WI per BW (Figure-2, p=0.047). The ratio of DMI and WI revealed the chronological phenomenon of the effect of high salinity water under our conditions. The ratio of DMI and WI during D16–21 from SW1.5 was significantly lower than that from SW0 (Figure-3, p<0.05). In this study, UV was determined from D8–21 of the experiment. High salinity in drinking water significantly increased daily UV (Figure-4, p=0.001). Significantly higher UV in SW1.5 cells was detected from D13–18 (p<0.05).

On the other hand, no effect of saline water on the bodyweight of goats was observed. Similarly, weight gain in the 1^st^ week after animals drank saline water did not differ between the groups (p=1.00, Table-4). However, weight gain in animals drinking saline water tended to decrease compared to those drinking freshwater in the 2^nd^ week of post-treatment (p=0.056, Table-4). Furthermore, plasma electrolytes and creatinine contents were similar between the groups from the pre-treatment (day 6) and treatment periods (day 14 and day 21) (p>0.05, Table-5). The concentration of urinary electrolytes was similar between the groups during the pre-treatment period, whereas the Na^+^ and Cl^-^ contents from SW1.5 were higher than those from SW0 during the treatment period (p<0.05, Table-6). Levels of urinary excretion (Uex) were similar between the groups during the pre-treatment period (day 1 and day 6). During the treatment period, the excretion of Na^+^ and Cl^-^ was significantly different between the groups. The Uex of Na^+^ and Cl^-^ from SW1.5 was higher than SW0 (p<0.05, Table-7). Finally, the Uex of K^+^ was different on day 14 but not on other days of study.

**Table 4:** Effects of high salinity in drinking water on body weight and weight gain.

Items	Period	Treatment		p-value

		SW0	SW1.5	
Body weight (kg/head)	Initial	22.54±0.26	22.86±0.57	0.62
	Wk_1_	23.36±0.44	23.68±0.65	0.69
	Wk_2_	24.16±0.37	24.08±0.74	0.93
	Average	23.76±0.40	23.88±0.69	0.88
Weight gain (g/head/day)	Wk_1_	117.14±41.50	117.14±19.38	1.00
	Wk_2_	114.29±15.65	57.14±20.20	0.0656
	Average	115.71±15.71	87.14±14.71	0.22

Treatment=SW0=Freshwater containing 0.127‰ TDS; SW1.5=High salinity water containing 15‰ TDS, SW=Seawater, TDS=Total Dissolved Solids

**Table 5:** Effects of high salinity in drinking water on plasma electrolytes and creatinine.

Items	Day	Treatment		p-value

		SW0	SW1.5	
Na^+^ (mmol/L)	D_6_	142.80±0.76	143.20±0.41	0.66
	D_14_	142.63±0.67	142.83±0.31	0.79
	D_21_	142.53±0.33	143.15±0.70	0.45
K^+^ (mmol/L)	D_6_	5.38±0.63	5.34±0.27	0.96
	D_14_	5.15±0.33	5.65±0.20	0.24
	D_21_	5.01±0.35	5.35±0.14	0.41
Cl^-^ (mmol/L)	D_6_	99.43±0.88	100.60±0.79	0.36
	D_14_	101.33±0.62	101.53±0.43	0.80
	D_21_	99.73±0.55	100.25±0.34	0.45
Creatinine (mmol/L)	D_6_	70.50±4.6	73.50±6.7	0.72
	D_14_	79.25±2.8	82.50±4.1	0.54
	D_21_	77.50±2.8	83.00±6.5	0.47

Treatment: SW0=Freshwater containing 0.127‰ TDS; SW1.5=High salinity water containing 15‰ TDS. D_6_=Pre-treatment period on day 6 of experiment; D_14_=Treatment period on day 14 of experiment; D_21_=Treatment period on day 21 of experiment. Na^+^=Sodium; K^+^=Potassium; Cl^-^=Chloride, SW=Seawater, TDS=Total Dissolved Solids

**Table 6:** Effects of high salinity in drinking water on urinary electrolyte concentrations.

Items	Day	Treatment		p-value

		SW0	SW1.5	
Na^+^ (mmol/L)	D_6_	21.18±1.0	23.10±0.89	0.21
	D_14_	22.40±0.86	134.30±3.5	0.001
	D_21_	24.58±1.1	108.60±12	0.001
K^+^ (mmol/L)	D_6_	137.60±13	134.50±11	0.86
	D_14_	142.40±12	135.40±14	0.72
	D_21_	159.90±14	145.80±16	0.52
Cl^-^ (mmol/L)	D_6_	181.60±21	194.90±25	0.70
	D_14_	189.80±16	286.30±29	0.03
	D_21_	192.10±22	247.50±10	0.06

Treatment: SW0=Freshwater containing 0.127‰ TDS; SW1.5=high salinity water containing 15‰ TDS. D_6_=Pre-treatment period on day 6 of experiment; D_14_=Treatment period on day 14 of experiment; D_21_=Treatment period on day 21 of experiment. Na^+^=Sodium; K^+^=Potassium; Cl^-^=Chloride, SW=Seawater, TDS=Total Dissolved Solids

**Table 7:** Effects of high salinity in drinking water on urinary electrolyte excretion.

Items	Day	Treatment		p-value

		SW0	SW1.5	
Uex Na^+^ (mmol/head/day)	D_6_	27.32±3.6	23.69±3.8	0.51
	D_14_	24.33±4.7	248.90±15	0.001
	D_21_	27.70±7.1	152.70±38	0.02
Uex K^+^ (mmol/head/day)	D_6_	172.80±13	137.10±21	0.21
	D_14_	152.60±26	249.70±25	0.03
	D_21_	168.20±22	194.10±22	0.44
Uex Cl^-^ (mmol/head/day)	D_6_	225.60±14	190.30±21	0.21
	D_14_	200.50±29	526.60±45	0.001
	D_21_	197.30±17	335.90±46	0.03

Treatment: SW0=Freshwater containing 0.127‰ TDS; SW1.5=High salinity water containing 15‰ TDS. D_6_=Pre-treatment period at day 6 of experiment; D_14_=Treatment period at day 14 of experiment; D_21_=Treatment period at day 21 of experiment. Na^+^=Sodium; K^+^=Potassium; Cl^-^=Chloride, Uex=Urinary electrolyte excretion, SW=Seawater, TDS=Total Dissolved Solids

## Discussion

The present study demonstrated that high salinity in water affects the drinking and eating behaviors of goats under tropical conditions. In addition, goats drinking water with high salinity exhibited a bigger UV. Furthermore, high salinity in water influenced urine output earlier than it influenced eating and drinking behaviors. These effects were related to an increase in the excretion capacity of both Na^+^ and Cl^-^.

High salinity in drinking water did not affect DMI and WI during the 1^st^ week of the treatment period, D8–15 (Figure-4). Similar results have been reported by previous studies. For example, Tsukahara *et al*. [15] found that brackish water containing 6900 mg TDS/L did not affect DMI in growing Boer and Spanish goats. Similarly, DMI was not significantly different among treatments when sheep drank water with saline levels ranging from 640 to 8326 mg TDS/L [16]. Moreover, some studies have reported that feed intake is negatively affected by increasing levels in saline water, and this result was similar to the previous studies in goats [5,17] and sheep [18]. On this note, the different responses observed in the animals may be due to different salt levels in water or the difference between species. The reduction in feed intake in animals that consumed water high in salinity was affected by a decrease in nutrient digestibility (DM and NDF) as observed in this study (data not shown) or ruminal function and passage rate [19].

In the 1^st^ week of treatment, daily WI was not affected by high salinity in the water, whereas daily WI tended to increase in the 2^nd^ week of the study. Thus, this indicated that saline water affected water consumption in Boer male crossbred goats in this study. In this regard, many studies found that WI increased as the salinity level increased. According to Mohammed [20], goats that drank water with 1.5% NaCl demonstrated an increased WI compared with those drinking freshwater. Similarly, Yousfi and Salem [18] reported a higher WI in sheep offered saline water with 11 g and 15 g of NaCl.

On the other hand, some studies found that WI was greater at low levels of salinity; however, when the saline level increased up to 2%, the water consumption decreased [21]. These results indicate that at low levels of saline, animals consume more water; conversely, when given high salinity water, animals decrease WI to avoid salt stress from the saline water [22]. From a behavioral point of view, the majority of drinking bouts are associated with eating, and the amount of WI is positively correlated with the quantity of food ingested [23]. However, the data from the present study revealed that animals drinking water with high salinity tended to have increased WI but maintained or decreased DMI as a result of a lower ratio of DMI per WI (Figure-4). Thus, this suggested the effect of salinity on drinking behavior. Interestingly, this behavioral change occurred later than the adaptation of the kidneys handling electrolyte load due to the intake of water high in salinity (see below).

Furthermore, the results of this study show that saline water did not affect BW throughout the experiment or weight gain during the 1^st^ week post-treatment. However, weight gain observed in the goats drinking highly saline water tended to decrease compared to those drinking freshwater in the 2^nd^ week post-treatment (p=0.056, Table-4). The lower weight gain in the high salinity group may be due to the lower DMI. Similar results were reported by Mdletshe *et al*. [17] who found that the weight gain in goats significantly decreased as the salinity levels increased due to the decrease in feed intake. However, a previous study observed that there was no effect on BW in sheep drinking water with 1% NaCl; conversely, a decrease in the BW of some animals receiving 1.5% NaCl in water was detected, and 2% NaCl resulted in decreased BW for all of the sheep [18].

On another note, plasma electrolytes were similar between the groups throughout the experiment and were within the normal ranges for healthy goats, as mentioned by Zoidis and Hadjigeorgiou [5] and Runa *et al*. [4]. Similarly, Nguyen *et al*. [9,10] found that dairy goats fed high levels of Na^+^ and K^+^ in their diet exhibited unchanged plasma concentrations of Na^+^ and K^+^. In this sense, the present study demonstrates that saline water has very high Na^+^ and Cl^-^ concentrations compared with freshwater (Table-1). However, goats from the SW1.5 group were still in the reference range and maintained constant plasma Na^+^ and Cl^-^ concentrations. The animals’ ability to tolerate various degrees of salt loads in drinking water may be related to kidney function [6]. Potter [7] suggested that sheep drinking water containing 1.3% NaCl appear to develop an adaptation for salt tolerance without any negative effects. This ability of sheep came from a renal adjustment that increased filtration and eliminated salt [6].

In the present experiment, UV and the excretion of Na^+^ and Cl^-^ measured in the SW1.5 group were greater than those in the SW0 group on day 14. It is noteworthy that these effects were more pronounced than those of drinking and eating behaviors. Thus, the results suggested that goats drinking saline water controlled the salt load by either excreting more UV and increasing the filtration rate or excreting more Na^+^ and Cl^-^ contents through urine (Tables-6 and 7). In addition, the chronological phenomenon also suggested the coupling of kidney function and behaviors. Furthermore, in this study, plasma and urinary K^+^ concentrations were not significantly affected by high salinity in drinking water. However, an increase in K^+^ excretion through urine was detected on day 14 but not on day 6 or day 21. This difference was mainly affected by the difference in UV on that day. Moreover, the study found that plasma K^+^ content was not affected by increasing NaCl levels in drinking water, while urinary K^+^ concentration increased with high NaCl in drinking water [5]. However, increases in plasma K^+^ concentration were found in goats [24] and sheep [6] when given saline water.

On another note, creatinine is a byproduct of muscle metabolism and is excreted by glomerular filtration. Thus, creatinine can be used as an indicator of renal function. In this study, the creatinine concentration in plasma remained unchanged between the groups and was at the reference level [23], indicating no adverse effects on kidney function due to goats consuming highly saline water. This result is similar to that reported by Runa *et al*. [24] when goats drank water with high salinity without an effect on plasma creatinine, whereas Zoidis and Hadjigeorgiou [5] reported that plasma creatinine levels increased with the high salinity in the water.

## Conclusion

Although this study has specific limitations, such as the limited number of animals, the results demonstrate that salinity influences drinking and eating behavior in goats under tropical countries. Furthermore, the kidneys’ responses to saline water were faster than behavioral responses such as drinking and eating behaviors. This adaptive mechanism in growing crossbred goats drinking saline water is activated by regulating water and salt balance by reducing the reabsorption of Na^+^ and Cl^-^ in the renal tubules and increasing excretion through urine. In addition, goats that drank highly saline water tended to experience decreased weight gain; however, they consumed more water than the control group. Therefore, this study concluded that crossbred male goats can tolerate 1.5% saline water from diluted SW for 2 weeks. However, more detailed experiments are required to determine the goats’ ability to respond to different levels of salinity in drinking water for a longer period.

## Authors’ Contributions

NT, NVH, NTN, and ST: Contributed to the conception and designed the study. NT, NVH, and NTN: Contributed reagents/materials/analysis tools. NT: Performed the animal experiments. NT and ST: Analyzed the data and performed the statistics. NT and ST: Wrote and revised the manuscript. All authors contributed to the final version of the manuscript and read and approved the final manuscript.
